# Enhanced Transdermal Delivery of rhHAPLN1 by Soluball^®^ Promotes Pericellular Matrix Stability and Keratinocyte Protection

**DOI:** 10.3390/pharmaceutics18080947

**Published:** 2026-07-31

**Authors:** Kyeong Hyeon Lee, Kang Min Kim, Ju Hyuk Han, Kyung Taek Oh, Dae Kyong Kim

**Affiliations:** 1 Department of Regulatory Pharmacy, Graduate School, Chung-Ang University, Seoul 06974, Republic of Korea; alex3188@naver.com; 2 HaplnScience Inc., Anyang 14058, Republic of Korea; 3 Silexn Technology Co., Ltd., Busan, 48547, Republic of Korea; 4 Department of Pharmaceutical Science and Technology, Kyungsung University, Busan 48434, Republic of Korea; 5 Brain Busan 21 Plus Research Group, Kyungsung University, Busan 48434, Republic of Korea

**Keywords:** rhHAPLN1, pericellular matrix, hyaluronan, microenvironment-targeted therapy, mesoporous silica, transdermal delivery, Soluball^®^

## Abstract

**Background/Objectives:** The pericellular matrix (PCM), a highly hydrated hyaluronan (HA)-rich extracellular structure surrounding keratinocytes, serves as a critical regulator of cellular protection, mechanobiological signaling, and epidermal microenvironmental homeostasis. Increasing evidence suggests that age- and stress-associated degradation of the HA-rich PCM contributes to impaired regenerative capacity and increased cellular vulnerability. Recombinant human hyaluronan and proteoglycan link protein 1 (rhHAPLN1) has emerged as a promising PCM-stabilizing biomolecule; however, its therapeutic application remains limited by poor skin permeability resulting from the barrier properties of the stratum corneum and the molecular size constraints governing hydrophilic macromolecule delivery. **Methods:** In the present study, we developed Soluball^®^, a dodecylamine-templated mesoporous silica-based carrier system designed to enhance the transdermal delivery of rhHAPLN1. **Results:** In vitro analyses demonstrated that rhHAPLN1 effectively preserved both the structural integrity and functional hydrodynamic volume of the PCM against hyaluronidase (HAdase)-induced degradation in HaCaT keratinocytes. Furthermore, rhHAPLN1 exhibited no significant cytotoxicity at concentrations up to 1 μg/mL and significantly enhanced keratinocyte proliferation under serum-free conditions. Physicochemical characterization revealed that Soluball^®^ possessed a relatively uniform particle size distribution (284.6 nm), a high specific surface area (1048 m^2^/g), and a mesoporous architecture with an average pore diameter of 3.8 nm, supporting efficient loading of hydrophilic biomolecules. Ex vivo permeation studies using human cadaver skin demonstrated that Soluball^®^-encapsulated rhHAPLN1 (H-S powder) significantly enhanced cumulative transdermal permeation compared with free rhHAPLN1 (5.54% vs. 0.88%, respectively). To further evaluate platform versatility, water-soluble Vitamin C was employed as a secondary model cargo. Vita-Soluball^®^ exhibited markedly enhanced permeation across both Strat-M^®^ artificial membranes and pig epidermis, achieving cumulative permeation values of 119.12 ± 9.38 μg/mL and 150.39 ± 29.20 μg/mL, respectively. **Conclusions:** Collectively, these findings suggest that rhHAPLN1 functions as an effective stabilizer of the HA-rich PCM and that Soluball^®^ enhances the transdermal delivery of hydrophilic biomolecules. Overall, Soluball^®^ may represent a promising transdermal delivery platform for hydrophilic biomolecules, although further in vivo validation is warranted.

## 1. Introduction

### 1.1. Biological Significance of the Pericellular Matrix

Recent advances in regenerative dermatology have shifted attention from the bulk extracellular matrix (ECM), traditionally focused on structural proteins such as collagen and elastin, toward the pericellular matrix (PCM), the immediate extracellular microenvironment surrounding cells [1]. The PCM is a highly hydrated, hyaluronan (HA)-rich structure associated with transmembrane receptors such as CD44 and plays an essential role in mechanotransduction, growth factor retention, and extracellular signaling [2,3,4]. Increasing evidence indicates that age-related deterioration of the HA-rich PCM impairs cellular resilience and regenerative capacity while increasing susceptibility to oxidative stress [5]. Accordingly, preservation of PCM integrity has emerged as a promising strategy for maintaining skin homeostasis and promoting tissue regeneration.

### 1.2. Biological Role of HAPLN1 in PCM Stabilization

Hyaluronan and proteoglycan link protein 1 (HAPLN1) is a key structural component responsible for the organization and stabilization of hyaluronan-rich extracellular matrices. By simultaneously binding hyaluronan and large proteoglycans such as versican and aggrecan, HAPLN1 promotes stable matrix assembly and preserves PCM integrity against hyaluronidase-mediated degradation [6,7]. Beyond its structural role, HAPLN1 contributes to extracellular matrix homeostasis and tissue regeneration, making recombinant human HAPLN1 (rhHAPLN1) a promising therapeutic biomolecule for maintaining PCM integrity and epidermal homeostasis [8,9]. Unlike conventional skin rejuvenation approaches focused on collagen stimulation, antioxidants, or growth factors, the present study proposes a PCM-targeted strategy that preserves the extracellular microenvironment. This is the first study to investigate mesoporous silica-mediated transdermal delivery of rhHAPLN1 for PCM stabilization in epidermal keratinocytes.

### 1.3. Limitations of Transdermal Delivery of rhHAPLN1

Despite its therapeutic potential, the clinical application of rhHAPLN1 remains challenging because of the barrier function of the stratum corneum (SC). Transdermal delivery of hydrophilic macromolecules, particularly those exceeding the “500 Dalton rule,” is generally limited [10]. As a relatively large hydrophilic protein, rhHAPLN1 exhibits poor passive skin permeation. Therefore, the development of a biocompatible carrier capable of improving the stability and transdermal delivery of rhHAPLN1 is essential for its regenerative and dermatological applications [11,12,13,14].

### 1.4. Mesoporous Silica-Based Delivery Systems and Soluball^®^

Mesoporous silica nanoparticles (MSNs) have been widely investigated as drug delivery platforms because of their high surface area, tunable pore architecture, and exceptional loading capacity for hydrophilic biomolecules [15,16]. However, conventional MSN synthesis frequently employs cetyltrimethylammonium bromide (CTAB) as a templating agent, and residual surfactants have been associated with cytotoxicity, membrane damage, and reduced biocompatibility [17]. To address these limitations, we developed Soluball^®^, a mesoporous silica carrier a dodecylamine-templated mesoporous silica carrier. Removal of the dodecylamine template during calcination contributes to improved biocompatibility, while optimized calcination conditions preserve silanol-rich surface structures that may facilitate hydrogen-bonding interactions with hydrophilic biomolecules [18]. Its highly porous architecture and large surface area may also support efficient encapsulation and stabilization of protein therapeutics. Accordingly, Soluball^®^ may provide a practical platform for enhancing the topical and transdermal delivery of PCM-stabilizing biomolecules such as rhHAPLN1. Recent advances in nanocarrier systems have highlighted the importance of engineering biomaterial platforms that improve the transport, stability, and biological activity of macromolecules. For example, Hou et al. demonstrated that lipid nanoparticle systems can effectively modulate biological functions through improved biomolecule delivery and microenvironmental regulation, underscoring the therapeutic potential of carrier-based delivery systems beyond conventional drug administration [9].

### 1.5. Objective of the Present Study

Based on these considerations, the present study aimed to develop a mesoporous silica-based transdermal delivery platform (Soluball^®^) for rhHAPLN1 and evaluate its ability to preserve PCM integrity, enhance skin permeation, and improve the delivery of hydrophilic biomolecules.

## 2. Materials and Methods

### 2.1. Chemicals and Reagents

Tetraethyl orthosilicate (TEOS, ≥98.0%) and dodecylamine (≥98.0%) were purchased from Sigma-Aldrich (St. Louis, MO, USA). Recombinant human hyaluronan and proteoglycan link protein 1 (rhHAPLN1) was provided by HaplnScience (Anyang, Republic of Korea). Mesoporous silica-based carrier (Soluball^®^) was supplied by Silexn Technology Co., Ltd. (Busan, Republic of Korea). Fluorescein isothiocyanate (FITC)-conjugated hyaluronan binding protein (HABP; Merck Millipore, Burlington, MA, USA) and DAPI (Thermo Fisher Scientific, Waltham, MA, USA) were used for visualization of the pericellular matrix (PCM) and cell nuclei, respectively. L-ascorbic acid (Vitamin C, ≥95.0%) was purchased from Sigma-Aldrich (St. Louis, MO, USA) and used for comparative transdermal permeation studies.

### 2.2. Cell Culture

HaCaT human keratinocytes were cultured in Dulbecco’s Modified Eagle Medium (DMEM; Welgene, Gyeongsan, Republic of Korea) supplemented with 10% heat-inactivated fetal bovine serum (FBS; Gibco, Grand Island, NY, USA) and 1% penicillin–streptomycin (P/S; Gibco, Grand Island, NY, USA). Cells were maintained at 37 °C in a humidified incubator containing 5% CO_2_.

### 2.3. Preparation and Characterization of Soluball^®^

Soluball^®^ was synthesized using a dodecylamine-templated sol–gel method. Briefly, dodecylamine was dissolved in an aqueous ethanol solution and used as a structure-directing template. TEOS was subsequently added to initiate silica condensation and particle formation. Following synthesis, the resulting silica particles were subjected to calcination for removal of the organic template. The physicochemical properties of Soluball^®^ were characterized using scanning electron microscopy (SEM; JSM-7610F, JEOL Ltd., Tokyo, Japan), Brunauer–Emmett–Teller (BET) surface area analysis (ASAP 2020, Micromeritics Instrument Corp., Norcross, GA, USA), Barrett–Joyner–Halenda (BJH) pore size distribution analysis, and dynamic light scattering (DLS; Zetasizer Nano ZS90, Malvern Panalytical, Malvern, UK) for particle size measurement.

### 2.4. Preparation of rhHAPLN1-Loaded Soluball^®^

rhHAPLN1-loaded Soluball^®^ (H-S powder) was prepared using a vacuum-assisted impregnation method. Briefly, rhHAPLN1 solution was mixed with Soluball^®^ particles and subjected to repeated vacuum cycles to facilitate protein incorporation into the mesoporous structure. The final loading concentration of rhHAPLN1 was adjusted to 0.1% (*w*/*w*). For comparative permeation studies, Vitamin C-loaded Soluball^®^ (Vita-Soluball) was prepared using the same method at a loading concentration of 20% (*w*/*w*).

Encapsulation efficiency and loading capacity were not quantitatively determined in the present study. Nevertheless, the enhanced transdermal permeation observed for H-S powder indirectly supports successful incorporation of rhHAPLN1 within the Soluball^®^ mesoporous carrier system.

### 2.5. Evaluation of PCM Stabilization

HaCaT cells were treated with rhHAPLN1 (1 µg/mL) prior to exposure to hyaluronidase (HAdase, 100 U/mL; Sigma-Aldrich, St. Louis, MO, USA) for 1 h. PCM morphology was visualized using confocal laser scanning microscopy (CLSM; LSM 800, Carl Zeiss, Oberkochen, Germany) following staining with FITC-HABP. Nuclear morphology was evaluated using DAPI staining. To evaluate the functional volume of the PCM, a red blood cell (RBC) exclusion assay was performed using sheep RBC suspension (1 × 10^8^ cells/mL) for 30 min. The exclusion halo area surrounding the cells was quantified using ImageJ version 1.54 (National Institutes of Health, Bethesda, MD, USA). Quantitative image analyses were performed using approximately 50 randomly selected cells from three independent experiments.

### 2.6. Cell Viability and Proliferation Assay

Cell viability and proliferative activity were evaluated using the Cell Counting Kit-8 (CCK-8) assay. HaCaT cells were treated with various concentrations of free rhHAPLN1 (1 ng/mL–10 µg/mL) or H-S powder for 24 h and 48 h under serum-containing and serum-free conditions. Cell viability was measured according to the manufacturer’s protocol.

### 2.7. Ex Vivo Transdermal Permeation Study

Transdermal permeation studies were performed using Franz diffusion cells under sink conditions. Human cadaver epidermal skin samples with a thickness of less than 0.1 mm and a diameter of 1.5 cm were used to evaluate the permeation behavior of free rhHAPLN1 and H-S powder over a 24 h period. Skin samples were visually inspected prior to use and handled in accordance with institutional ethical guidelines. The receptor chamber was filled with phosphate-buffered saline (PBS, pH 7.4) and maintained at 32 °C under continuous magnetic stirring throughout the experiment. Samples were collected at predetermined intervals during the 24 h permeation study, and cumulative permeation (%) was calculated based on the total amount of rhHAPLN1 detected in the receptor chamber relative to the initially applied dose. To further investigate the versatility of the Soluball^®^ delivery platform, Strat-M^®^ artificial membrane and porcine epidermis models were employed to compare the cumulative permeation and permeability coefficient (Kp) of free Vitamin C and Vita-Soluball^®^ formulations.

### 2.8. Statistical Analysis

All experiments were performed using three independent biological replicates. Data are presented as mean ± standard deviation (SD). Statistical analyses were conducted using GraphPad Prism version 10.0 (GraphPad Software, San Diego, CA, USA). Differences among groups were analyzed using one-way analysis of variance (ANOVA) followed by Tukey’s post hoc test. Normality assumptions were evaluated prior to statistical testing. A *p*-value less than 0.05 was considered statistically significant.

## 3. Results

### 3.1. Protective Effect of rhHAPLN1 on Pericellular Matrix Stability in HaCaT Keratinocytes

**Figure 1:**
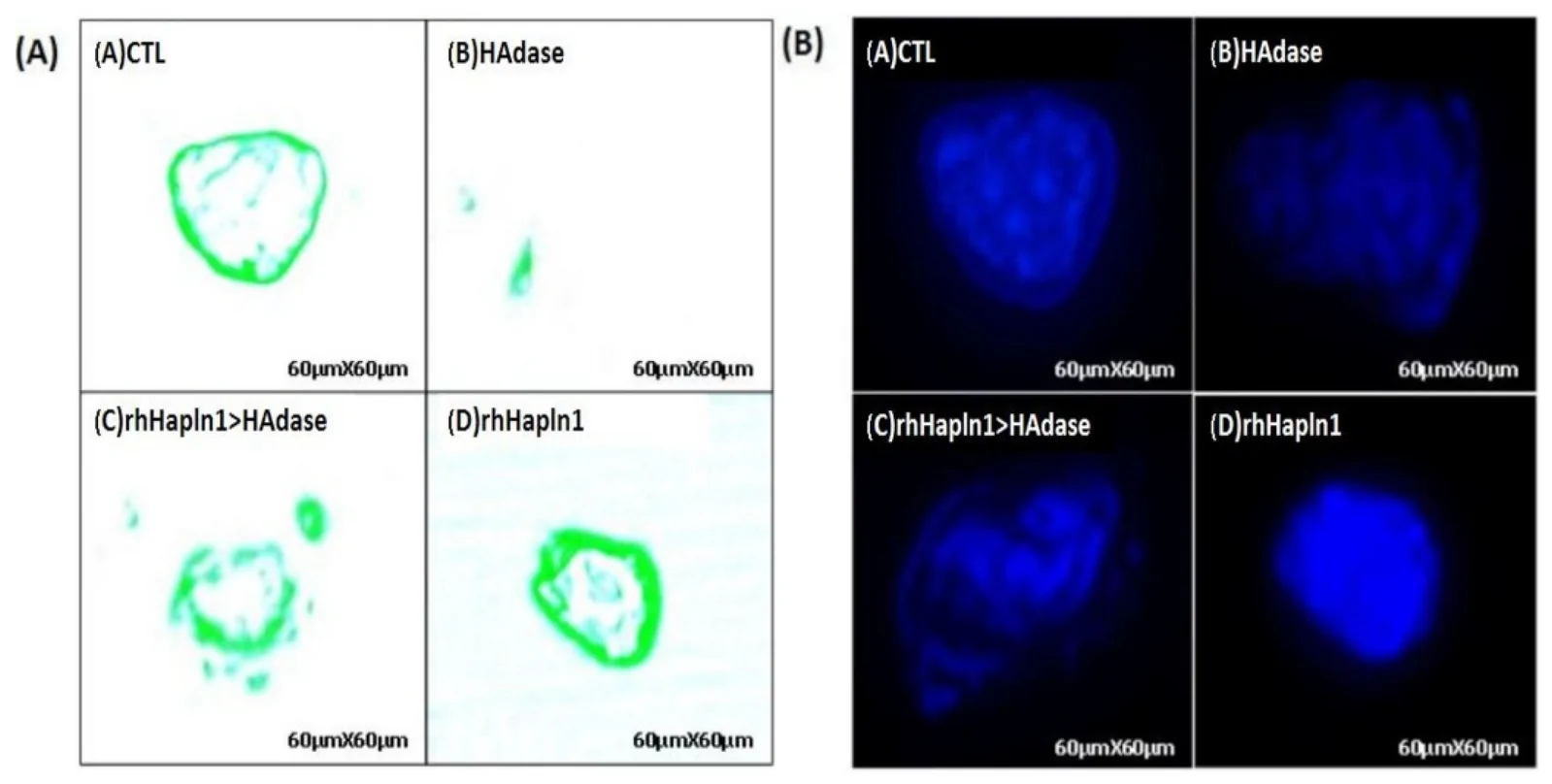
Protective Effect of rhHAPLN1 on Pericellular Matrix (PCM) Integrity in HaCaT Keratinocytes. (**A**) Visualization of the HA-rich pericellular matrix (PCM) by FITC-HABP staining. Confocal laser scanning microscopy (CLSM) images of HaCaT keratinocytes stained with FITC-conjugated hyaluronan-binding protein (FITC-HABP, green) to visualize the HA-rich PCM. The untreated control exhibited a continuous PCM, whereas hyaluronidase (HAdase, 100 U/mL) markedly degraded the PCM. Pretreatment with rhHAPLN1 (1 µg/mL) preserved PCM integrity following HAdase exposure. (**B**) Evaluation of cellular morphology by DAPI staining. DAPI staining (blue) was used to visualize cell nuclei and assess cellular morphology. rhHAPLN1-treated cells maintained normal nuclear morphology and cellular organization following HAdase treatment. Representative images were selected from approximately 50 cells per experimental group. Scale bar = 60 µm.

The pericellular matrix (PCM), a hyaluronan (HA)-rich extracellular structure surrounding keratinocytes, plays a critical role in maintaining cellular protection, biomechanical buffering, and microenvironmental homeostasis. To investigate whether recombinant human hyaluronan and proteoglycan link protein 1 (rhHAPLN1) protects the PCM against enzymatic degradation, morphological changes in the HA-rich matrix were analyzed by confocal laser scanning microscopy (CLSM) following FITC-HABP staining. In the untreated control group, HaCaT keratinocytes exhibited a dense and continuous HA-rich PCM surrounding the cell membrane, characterized by strong FITC-HABP fluorescence intensity. In contrast, exposure to hyaluronidase (HAdase, 100 U/mL) for 1 h markedly reduced FITC-HABP fluorescence, indicating substantial degradation and collapse of the PCM structure. Notably, pre-treatment with rhHAPLN1 (1 µg/mL) significantly preserved the HA-rich extracellular matrix despite subsequent HAdase exposure, maintaining relatively continuous and intense fluorescence patterns surrounding the cells. Nuclear morphology was further evaluated by DAPI staining to assess cellular integrity under PCM-degrading conditions. In contrast, cells pre-treated with rhHAPLN1 maintained relatively intact nuclear morphology and preserved cellular organization following HAdase exposure. These findings indicate that preservation of PCM integrity by rhHAPLN1 may contribute not only to extracellular matrix stabilization but also to the maintenance of overall cellular microenvironmental stability under enzymatic stress conditions. The representative CLSM images are shown in Figure 1. Representative images were selected from approximately 50 randomly selected cells obtained from three independent experiments.

### 3.2. Quantitative Evaluation of PCM Volume by RBC Exclusion Assay

**Figure 2:**
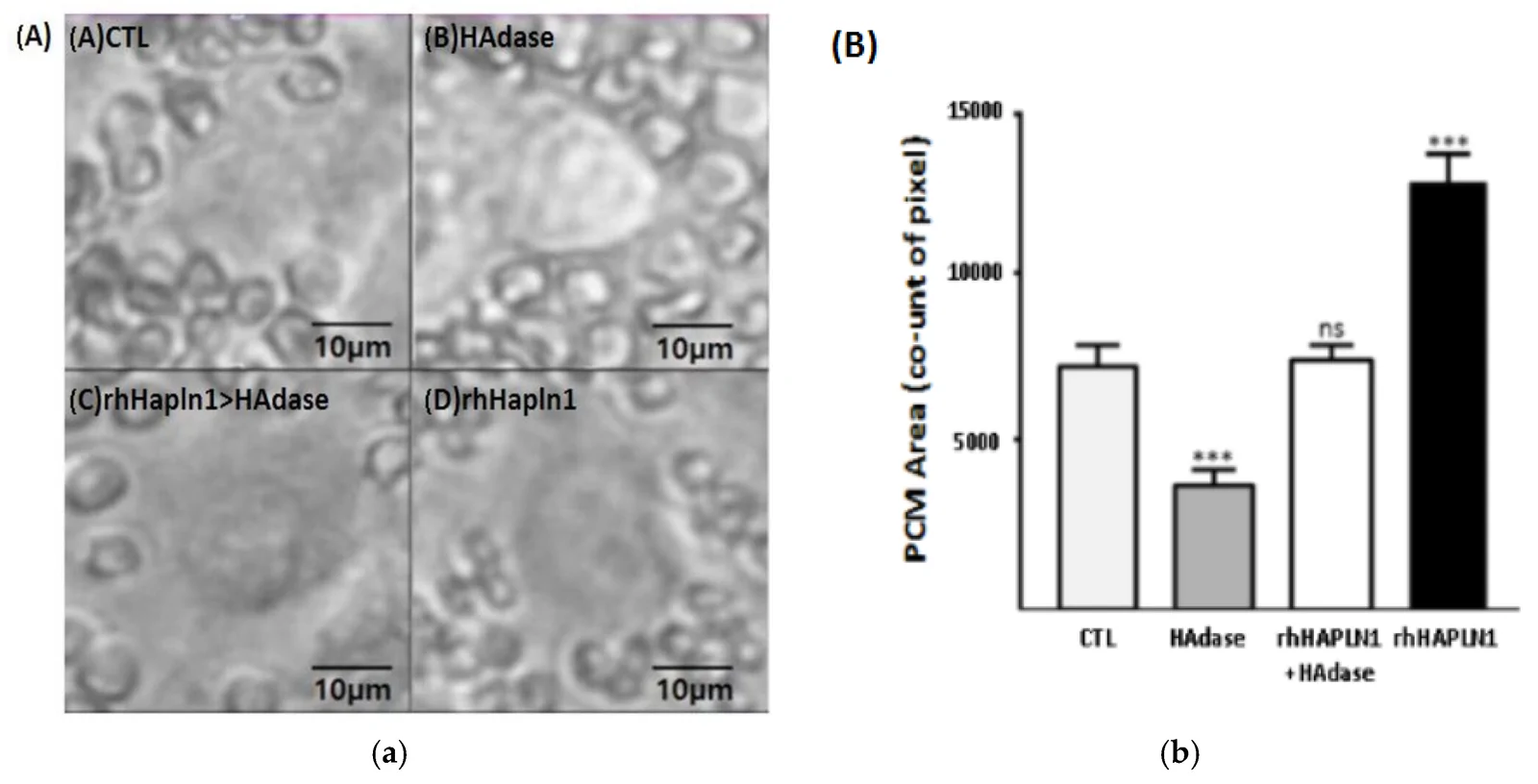
Quantitative Evaluation of PCM Volume by RBC Exclusion Assay. (**A**) Representative images of the RBC exclusion assay in HaCaT keratinocytes. Bright-field microscopy images showing exclusion of sheep red blood cells (RBCs; 1 × 10^8^ cells/mL, 30 min) by the hyaluronan-rich pericellular matrix (PCM). The untreated control exhibited a distinct exclusion halo, whereas hyaluronidase (HAdase) treatment reduced halo formation. Pretreatment with rhHAPLN1 (1 µg/mL) preserved the exclusion halo following HAdase exposure. (**B**) Quantitative analysis of exclusion halo area. Halo areas were quantified by pixel-based image analysis to evaluate PCM volume surrounding HaCaT keratinocytes. Representative analyses were performed using approximately 50 cells per experimental group. Statistical significance was determined using one-way ANOVA followed by Tukey’s post hoc test (ns, not significant; *** *p* < 0.001).

To further assess the functional preservation of the pericellular matrix (PCM), a red blood cell (RBC) exclusion assay was performed to quantitatively evaluate the hydrodynamic matrix volume surrounding HaCaT keratinocytes (Figure 2). In the untreated control group, HaCaT keratinocytes exhibited a prominent and well-defined exclusion zone, reflecting the presence of an intact and functionally hydrated PCM. In contrast, treatment with hyaluronidase (HAdase) markedly reduced the halo area, indicating substantial degradation and collapse of the HA-rich pericellular structure. Notably, cells pre-treated with rhHAPLN1 (1 µg/mL) retained significantly larger exclusion zones following HAdase exposure compared with the HAdase-only group (*p* < 0.001). Quantitative image analysis confirmed that rhHAPLN1 significantly preserved the functional hydrodynamic volume of the PCM under enzymatic stress by attenuating the HAdase-induced reduction in halo area. Halo area measurements were obtained from approximately 50 cells per experimental group.

### 3.3. Effects of rhHAPLN1 on Cell Viability and Proliferation in HaCaT Keratinocytes

**Figure 3:**
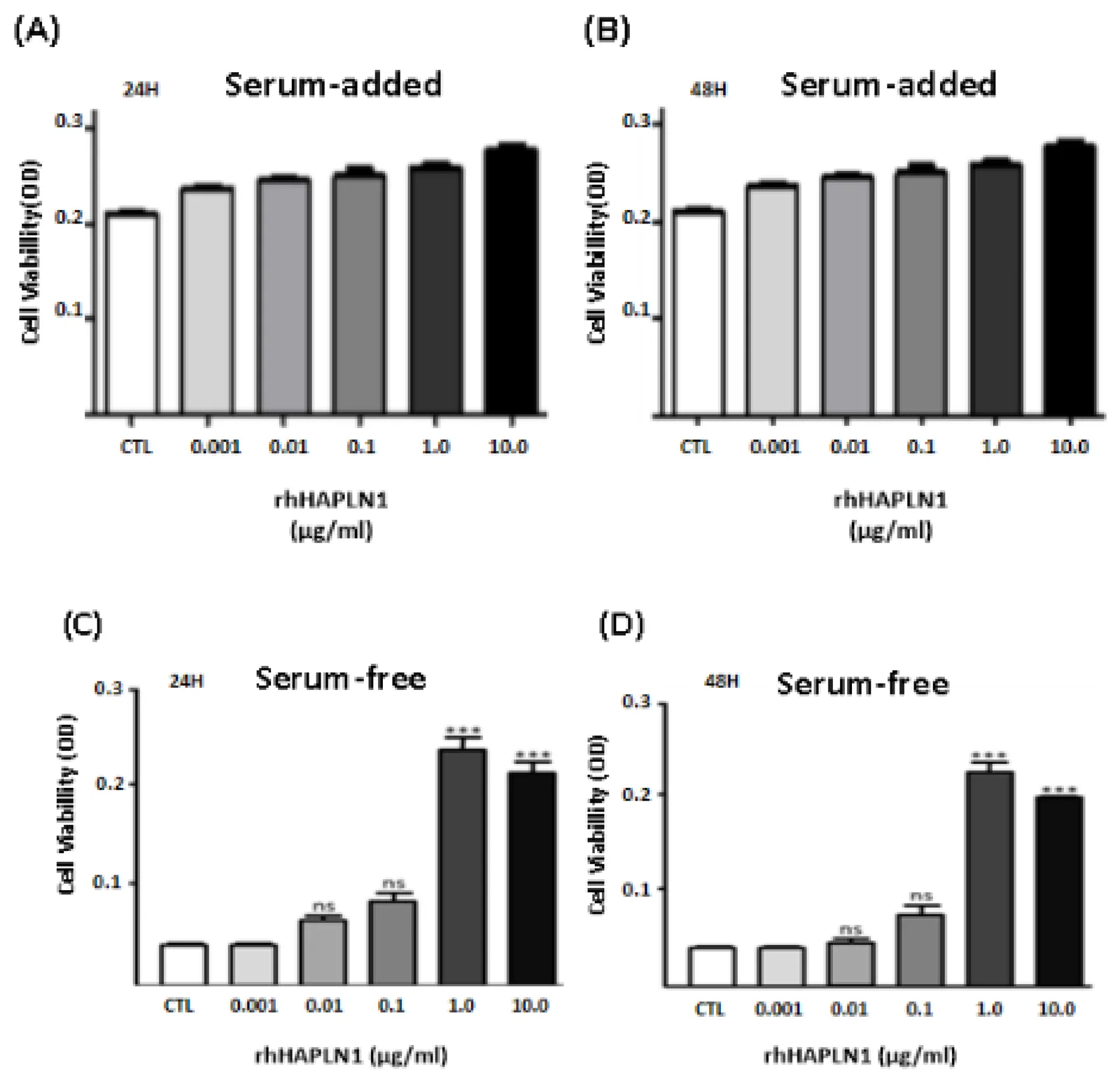
Effects of rhHAPLN1 on Cell Viability and Proliferation in HaCaT Keratinocytes. (**A**,**B**) Evaluation of cell viability and proliferative activity following rhHAPLN1 treatment. HaCaT keratinocytes were treated with rhHAPLN1 (1 ng/mL–10 µg/mL) for 24 or 48 h. Cell viability and proliferative activity were evaluated using the CCK-8 assay under serum-containing and serum-free conditions, respectively. (**C**,**D**) Effects of rhHAPLN1 on HaCaT keratinocytes under different culture conditions. rhHAPLN1 exhibited no significant cytotoxicity under serum-containing conditions and promoted concentration-dependent proliferation under serum-free conditions. Data are presented as mean ± SD from three independent biological experiments. Statistical significance was determined using one-way ANOVA followed by Tukey’s post hoc test (ns, not significant, *** *p* < 0.001).

The biocompatibility of recombinant human hyaluronan and proteoglycan link protein 1 (rhHAPLN1) was evaluated in HaCaT keratinocytes using the CCK-8 assay following 24 h and 48 h treatment periods. Across the tested concentration range (1 ng/mL to 10 µg/mL), rhHAPLN1 exhibited no significant cytotoxicity under serum-containing culture conditions, and overall cell viability remained comparable to that of the untreated control group. These results indicate that rhHAPLN1 is well tolerated by HaCaT keratinocytes over a broad concentration range. To further investigate whether rhHAPLN1 influences keratinocyte growth behavior, cells were cultured under serum-free conditions to minimize the effects of exogenous growth factors. Under these conditions, rhHAPLN1 treatment induced a concentration-dependent increase in HaCaT cell proliferation. Notably, treatment with the highest concentration of rhHAPLN1 resulted in approximately a 5.5-fold increase in proliferative activity compared with the serum-free control group. Collectively, the results support the potential utility of rhHAPLN1 as a biologically active component for maintaining epidermal cellular activity and microenvironmental stability (Figure 3).

### 3.4. Physicochemical Characterization of Soluball^®^

**Figure 4:**
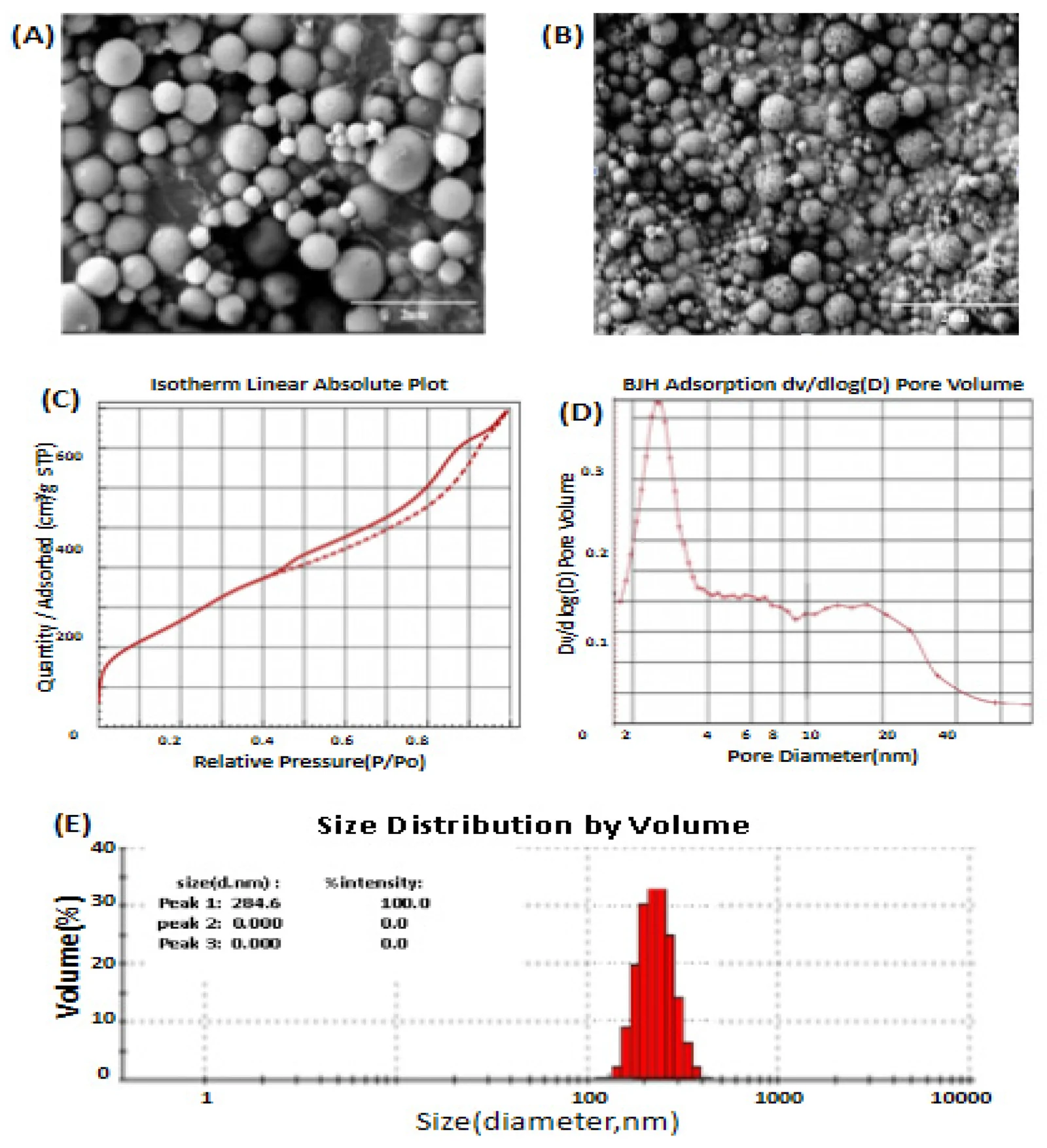
Physicochemical Characterization of Soluball^®^. (**A**,**B**) Scanning electron microscopy (SEM) images of Soluball^®^. SEM images showing the porous mesostructured silica morphology of Soluball^®^ following calcination. The particles exhibited a well-organized mesoporous architecture with abundant nanoscale pore structures distributed across the particle surface. (**C**) BET surface area analysis. Nitrogen adsorption–desorption isotherms analyzed using the Brunauer–Emmett–Teller (BET) method demonstrated that Soluball^®^ possessed a high specific surface area of 1048 m^2^/g, indicating substantial internal surface availability for biomolecular encapsulation. (**D**) BJH pore size distribution analysis. Barrett–Joyner–Halenda (BJH) analysis revealed a total pore volume of 1.21 cm^3^/g with an average pore diameter of 3.8 nm, confirming the presence of a highly developed mesoporous framework. (**E**) Dynamic light scattering (DLS) analysis. DLS measurements demonstrated an average hydrodynamic particle diameter of 284.6 nm with relatively uniform particle distribution, indicating favorable colloidal dispersion characteristics of the Soluball^®^ carrier system.

The physicochemical properties of the Soluball^®^ carrier system were comprehensively characterized using scanning electron microscopy (SEM), Brunauer–Emmett–Teller (BET) surface area analysis, Barrett–Joyner–Halenda (BJH) pore distribution analysis, and dynamic light scattering (DLS). SEM analysis revealed that Soluball^®^ exhibited a well-defined porous mesostructured silica morphology following calcination, indicating successful formation of the mesoporous framework. The particles displayed a relatively homogeneous surface architecture with abundant nanoscale pore structures, consistent with the intended mesoporous silica design. Nitrogen adsorption–desorption analysis based on the BET method demonstrated that Soluball^®^ possessed a high specific surface area of 1048 m^2^/g, suggesting substantial internal surface availability for biomolecular loading. Furthermore, BJH pore size distribution analysis revealed a total pore volume of 1.21 cm^3^/g and an average pore diameter of 3.8 nm, indicating the presence of a highly developed mesoporous network suitable for the encapsulation of hydrophilic biomolecules. Dynamic light scattering (DLS) analysis showed that Soluball^®^ exhibited an average hydrodynamic particle diameter of 284.6 nm with a relatively uniform particle size distribution, supporting the colloidal stability and dispersion uniformity of the carrier system. Collectively, these results indicate that Soluball^®^ possesses favorable mesoporous characteristics for the encapsulation and transdermal delivery of hydrophilic biomolecules (Figure 4).

### 3.5. Biocompatibility and Ex Vivo Transdermal Permeation of H-S Powder

**Figure 5:**
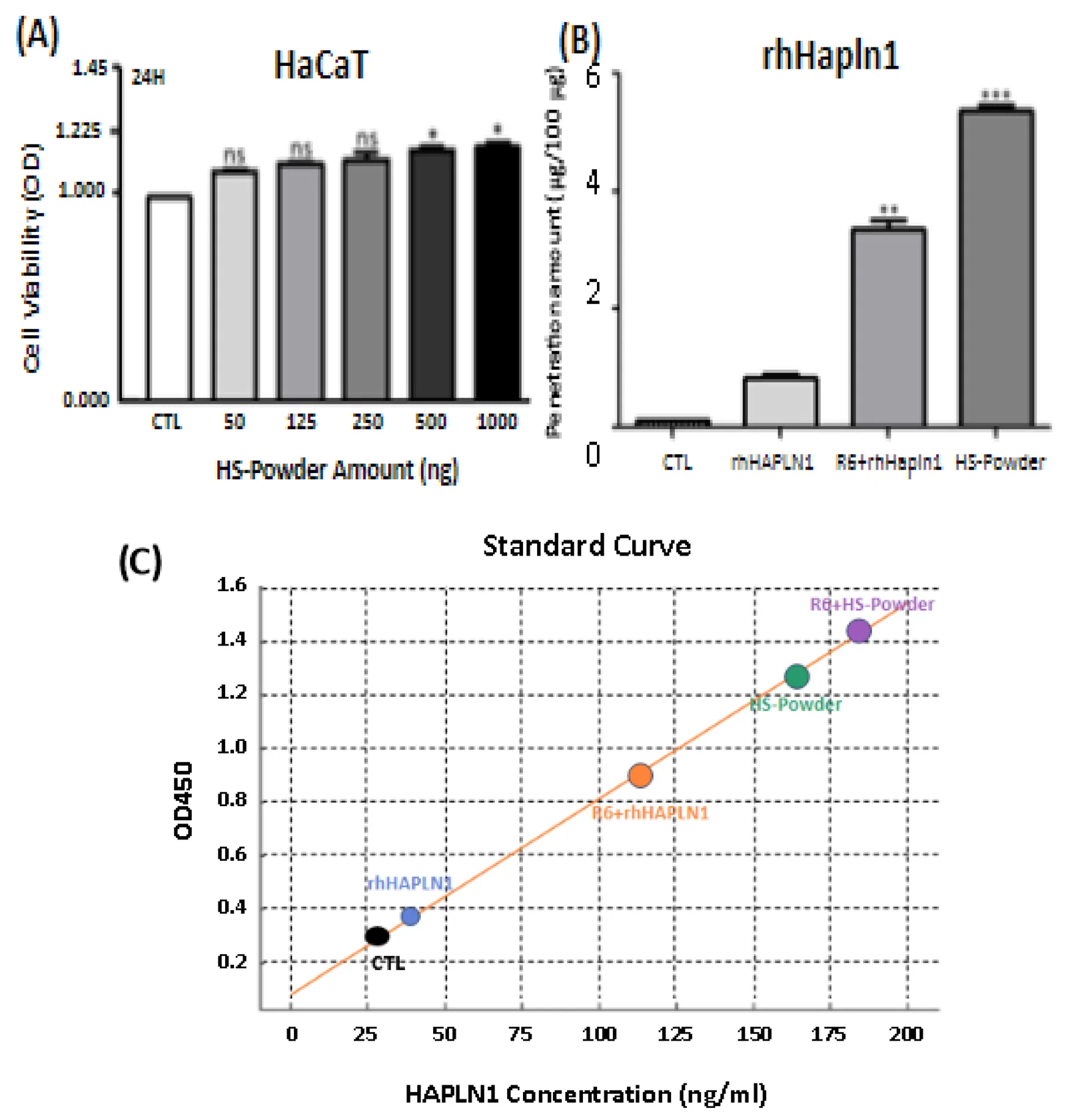
Biocompatibility and Ex Vivo Transdermal Permeation of H-S Powder. (**A**) Biocompatibility evaluation of H-S powder in HaCaT keratinocytes. Cell viability of HaCaT keratinocytes treated with rhHAPLN1-loaded Soluball^®^ (H-S powder; 0.1% *w*/*w* loading) for 24 h was evaluated using the CCK-8 assay. H-S powder maintained high cellular viability at concentrations up to 1 μg/mL, indicating favorable biocompatibility of the formulation. (**B**) Ex vivo transdermal permeation profiles of free rhHAPLN1 and H-S powder. Transdermal permeation studies were performed using Franz diffusion cells equipped with human cadaver skin over a 24 h period. Free rhHAPLN1 exhibited limited cumulative permeation (0.88%) across the skin membrane, whereas H-S powder significantly enhanced transdermal permeation, reaching 5.54% under identical experimental conditions. Data are presented as mean ± SD from three independent biological experiments. Statistical significance was determined using one-way ANOVA followed by Tukey’s post hoc test (ns, not significant, * *p* < 0.05, ** *p* < 0.01, *** *p* < 0.001). (**C**) Standard curve for rhHAPLN1 quantification. Standard calibration curve used for quantitative determination of rhHAPLN1 concentrations during ex vivo permeation analysis.

rhHAPLN1-loaded Soluball^®^ (H-S powder) was prepared using a vacuum-assisted impregnation method with a final rhHAPLN1 loading content of 0.1% (*w*/*w*). To evaluate the biological safety of the formulation, the biocompatibility of H-S powder was assessed in HaCaT keratinocytes using the CCK-8 assay. Under the tested conditions, H-S powder maintained high cell viability at concentrations up to 1 µg/mL, indicating that incorporation of rhHAPLN1 into the Soluball^®^ carrier system did not induce significant cytotoxicity and retained favorable cellular compatibility. To further investigate the transdermal delivery capability of the formulation, ex vivo permeation studies were conducted using Franz diffusion cells equipped with human cadaver skin over a 24 h period. Free rhHAPLN1 exhibited minimal cumulative permeation across the skin membrane, reaching only 0.88%, consistent with the limited passive diffusion typically observed for hydrophilic macromolecules. In contrast, H-S powder demonstrated substantially enhanced transdermal permeation, achieving a cumulative permeation rate of 5.54% under identical experimental conditions. These findings indicate that H-S powder significantly enhanced the transdermal permeation of rhHAPLN1 compared with free rhHAPLN1. Although the enhanced permeation may be related to the high surface area and mesoporous architecture of Soluball^®^, the underlying mechanism has not been directly investigated and therefore remains hypothetical (Figure 5).

### 3.6. Transdermal Permeation of Vitamin C Using Vita-Soluball^®^

**Figure 6:**
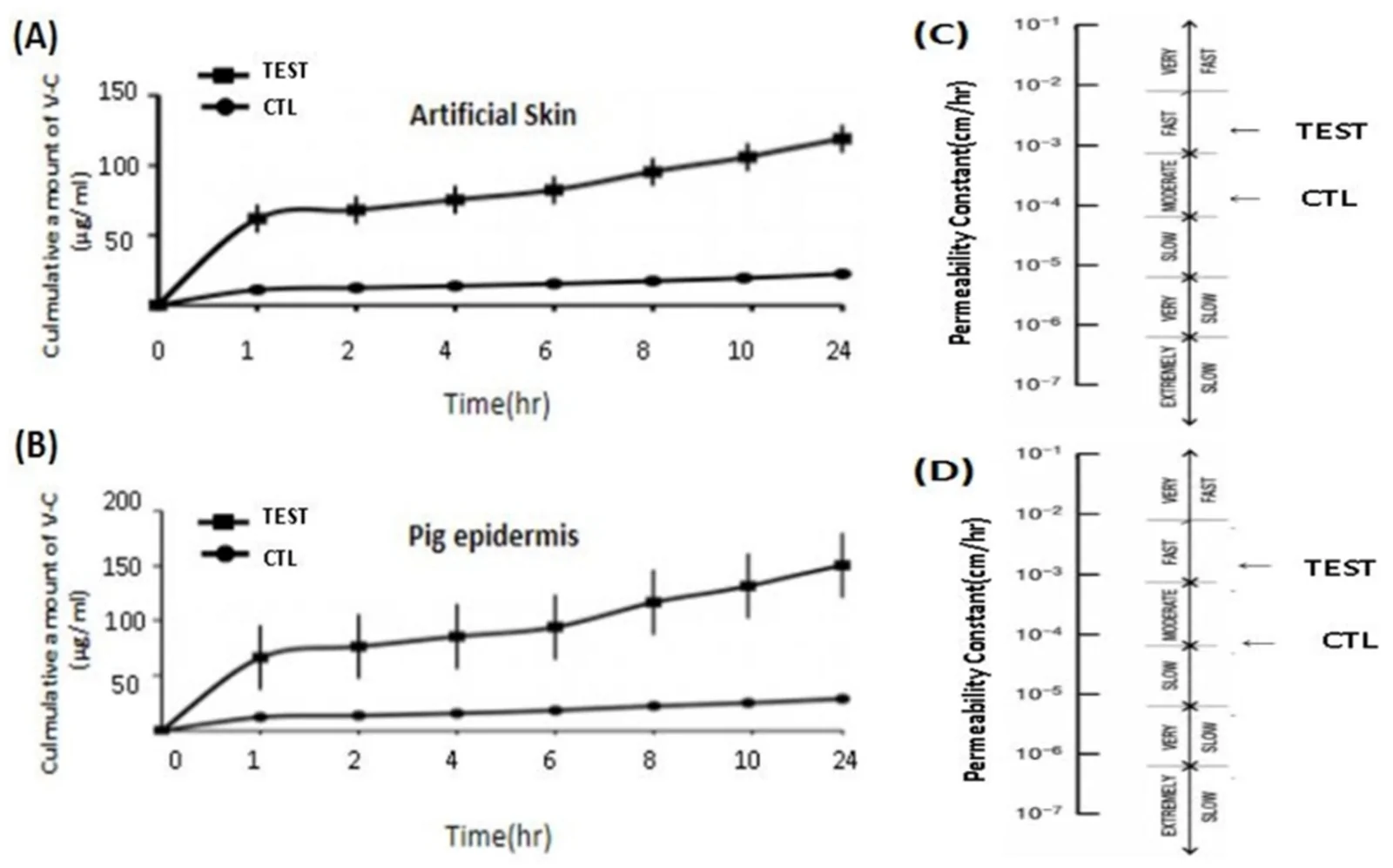
Transdermal Permeation of Water-Soluble Vitamin C Using Vita-Soluball^®^. (**A**) In vitro transdermal permeation across artificial skin membrane (Strat-M^®^ membrane). Transdermal permeation profiles of water-soluble Vitamin C across Strat-M^®^ artificial skin membranes over a 24 h period. Vita-Soluball^®^ demonstrated significantly higher cumulative permeation (119.12 ± 9.38 μg/mL) compared with free Vitamin C (22.21 ± 1.42 μg/mL). (**B**) Permeability coefficient (Kp) analysis in the artificial membrane model. Permeability coefficients were calculated according to the Marzulli classification system. Vita-Soluball^®^ exhibited an increased permeability coefficient (15.10 × 10^−4^ cm/h) compared with free Vitamin C (2.86 × 10^−4^ cm/h), corresponding to a shift from “Moderate” to “Fast” permeation status. (**C**) Ex vivo transdermal permeation across pig epidermis. Ex vivo permeation profiles of water-soluble Vitamin C across pig epidermal tissue over 24 h. Vita-Soluball^®^ showed substantially enhanced cumulative permeation (150.39 ± 29.20 μg/mL) compared with free Vitamin C (28.98 ± 1.27 μg/mL). (**D**) Permeability coefficient (Kp) analysis in the pig epidermis model. Vita-Soluball^®^ demonstrated a markedly increased permeability coefficient (20.48 × 10^−4^ cm/h) compared with free Vitamin C (4.03 × 10^−4^ cm/h), corresponding to “Fast” permeation status according to the Marzulli classification. Data are presented as mean ± SD from three independent experiments. Statistical significance was determined using one-way ANOVA followed by Tukey’s post hoc test.

To further investigate the broader applicability of the Soluball^®^ delivery platform for hydrophilic compounds, water-soluble Vitamin C was encapsulated into Soluball^®^ at a loading concentration of 20% (*w*/*w*) to generate the Vita-Soluball^®^ formulation. Transdermal permeation efficiency was evaluated using both artificial skin membrane models and ex vivo epidermal tissue models. In the artificial skin membrane model (Strat-M^®^ membrane), Vita-Soluball^®^ exhibited markedly enhanced transdermal permeation compared with free Vitamin C over a 24 h period. The cumulative permeation of Vita-Soluball^®^ reached 119.12 ± 9.38 μg/mL, whereas free Vitamin C showed substantially lower permeation (22.21 ± 1.42 μg/mL). Consistent with these findings, the calculated permeability coefficient (Kp) increased from 2.86 × 10^−4^ cm/h for free Vitamin C to 15.10 × 10^−4^ cm/h for Vita-Soluball^®^. According to the Marzulli classification system, this increase corresponded to a transition from “Moderate” to “Fast” permeation status. Comparable enhancement effects were observed in the ex vivo pig epidermis model. Vita-Soluball^®^ demonstrated significantly higher cumulative permeation than free Vitamin C over the 24 h experimental period. In addition, the permeability coefficient increased to 20.48 × 10^−4^ cm/h, corresponding to “Fast” permeation behavior according to the Marzulli classification. These results collectively indicate that the Soluball^®^ mesoporous carrier system effectively enhances the transdermal permeation efficiency of hydrophilic small molecules across both artificial and biologically derived skin barriers (Figure 6).

## 4. Discussion

### 4.1. Role of rhHAPLN1 in Pericellular Matrix Stabilization

The pericellular matrix (PCM) has increasingly emerged as a critical regulator of the cellular microenvironment, influencing mechanobiological signaling, tissue homeostasis, and regenerative responses in the skin [2,5]. Unlike the bulk extracellular matrix (ECM), the PCM forms an immediate HA-rich interface surrounding cells and functions as a dynamic platform for hydration buffering, receptor organization, growth factor retention, and environmental protection. Growing evidence suggests that deterioration of the HA-rich PCM during aging and chronic stress contributes to impaired cellular resilience and reduced regenerative capacity. In the present study, rhHAPLN1 significantly preserved PCM integrity in HaCaT keratinocytes under HAdase-induced degradative conditions, as demonstrated by both CLSM visualization and RBC exclusion assays. Together, these complementary approaches demonstrated that rhHAPLN1 preserved both the structural architecture and functional hydrodynamic volume of the HA-rich PCM. Because hydration-dependent matrix volume is a key determinant of PCM functionality, maintenance of the exclusion halo suggests preservation of biologically relevant matrix properties rather than merely retention of HA staining signals. HAPLN1 is known to stabilize extracellular matrix organization through simultaneous interactions with HA and proteoglycans such as versican and aggrecan [7]. The present findings support the concept that rhHAPLN1 functions as a molecular stabilizer of HA-rich pericellular assemblies and may protect the PCM from enzymatic disruption. Beyond structural stabilization, preservation of PCM integrity may also influence cellular functioning beyond structural maintenance. HA-rich PCM structures participate in the spatial organization of CD44 receptors, modulation of mechanotransduction, and regulation of extracellular signaling events. Disruption of these structures has been associated with impaired cellular protection, altered stress responses, and compromised tissue homeostasis [5]. Interestingly, rhHAPLN1 also promoted keratinocyte proliferation under serum-free conditions. Although the precise molecular mechanisms remain to be elucidated, stabilization of the PCM may create a favorable microenvironment that supports cellular survival and regenerative activity through maintenance of extracellular hydration, receptor clustering, and growth factor accessibility. Previous studies have implicated HA-rich pericellular matrices in CD44-mediated signaling pathways involved in cellular adaptation and tissue repair [19]. Collectively, these findings suggest that rhHAPLN1 may function not only as a structural matrix protein but also as a microenvironment-modulating biomolecule capable of supporting epidermal homeostasis and regenerative responses.

### 4.2. Physicochemical Characteristics and Biocompatibility of Soluball^®^

Mesoporous silica nanoparticles (MSNs) have attracted considerable interest as drug delivery platforms because of their high loading capacity and tunable structural characteristics [15]. However, concerns regarding residual surfactants and associated cytotoxicity remain important limitations of conventional CTAB-based MSN systems [17]. To address these challenges, Soluball^®^ was synthesized using a dodecylamine-templated sol–gel process designed to enhance biocompatibility while preserving favorable mesoporous characteristics. SEM, BET, BJH, and DLS analyses demonstrated that Soluball^®^ possesses a highly developed mesoporous framework with a large surface area (1048 m^2^/g), substantial pore volume, and uniform particle distribution. These structural features are advantageous for the encapsulation of hydrophilic biomolecules, which are often difficult to load efficiently into conventional carrier systems. Although the average pore diameter determined by BJH analysis was approximately 3.8 nm, this value primarily represents the internal mesoporous region rather than the pore entrances. Soluball^®^ possesses a funnel-like pore architecture with substantially larger pore openings (>15 nm) at the particle surface, which may facilitate the incorporation of relatively large biomolecules such as rhHAPLN1. Following adsorption at the pore openings, hydrogen-bonding and electrostatic interactions with silanol-rich silica surfaces may facilitate the incorporation and stabilization of rhHAPLN1 within the mesoporous framework, although the precise loading mechanism remains to be experimentally verified. Preservation of silanol-rich surface chemistry following calcination may also contribute to the loading stability and dispersion of hydrophilic compounds [18].

Although the precise loading mechanism was not directly investigated, these physicochemical characteristics likely contribute to the efficient incorporation and stabilization of rhHAPLN1 within the Soluball^®^ framework. Importantly, H-S powder maintained favorable cellular viability in HaCaT keratinocytes, indicating that incorporation of rhHAPLN1 into the carrier system did not induce significant cytotoxicity. These findings suggest that Soluball^®^ may provide a biocompatible alternative to conventional MSN formulations for topical and transdermal applications. However, the release kinetics of rhHAPLN1 from Soluball^®^ were not investigated in the present study. Future studies should evaluate sustained release profiles and release mechanisms to further characterize the functionality of this delivery platform. Future studies employing circular dichroism spectroscopy, SDS–PAGE, or protein stability assays would provide additional confirmation.

### 4.3. Transdermal Permeation of rhHAPLN1 Using Soluball^®^

One of the major barriers to the therapeutic application of protein-based biomolecules is their limited ability to penetrate the stratum corneum. This challenge is particularly pronounced for hydrophilic macromolecules such as rhHAPLN1, whose molecular size and physicochemical properties severely restrict passive skin permeation [10,14]. In the present study, Soluball^®^-encapsulated rhHAPLN1 achieved approximately six-fold greater cumulative permeation across human cadaver skin compared with free rhHAPLN1. Although the absolute permeation remained relatively modest, the magnitude of enhancement is noteworthy considering the intrinsic limitations associated with transdermal delivery of protein therapeutics. These findings suggest that mesoporous silica-based encapsulation may partially overcome the permeability constraints typically imposed by the skin barrier. The enhanced permeation may be associated with improved surface hydration, cargo dispersion, and follicular transport pathways; however, these mechanisms remain hypothetical and require further investigation. Nanoparticles within the submicron size range have been reported to access follicular and appendageal transport pathways, which can serve as alternative routes for delivery across the skin barrier [20]. Mesoporous silica particles may influence local hydration dynamics and modify interfacial interactions between cargo molecules and epidermal lipid structures. Encapsulation may also improve dispersion stability and maintain favorable concentration gradients at the skin surface, thereby facilitating transport. Future studies employing fluorescently labeled rhHAPLN1, follicular localization analyses, and in vivo biodistribution assessments will be valuable for defining the dominant pathways involved in delivery enhancement. Compared with previously reported transdermal strategies for hydrophilic macromolecules, including lipid nanoparticles, microneedles, and deformable vesicles, Soluball^®^ offers the advantages of high surface area, mesoporous architecture, and favorable biocompatibility. Although Strat-M^®^ membranes provide a convenient surrogate for human skin, they do not fully reproduce the structural complexity and biochemical composition of native tissue. Therefore, interpretation of artificial membrane permeation data should be complemented with biologically relevant skin models.

### 4.4. Applicability of the Soluball^®^ Platform for Hydrophilic Compounds

To explore whether Soluball^®^ possesses broader applicability beyond rhHAPLN1 delivery, Vitamin C was employed as a proof-of-concept hydrophilic cargo molecule. Vita-Soluball^®^ demonstrated substantially enhanced permeation across both artificial skin membranes and ex vivo pig epidermis compared with free Vitamin C. Moreover, permeability coefficient analyses revealed a transition from “Moderate” to “Fast” permeation according to the Marzulli classification system. The observation that both rhHAPLN1 and Vitamin C exhibited improved delivery following encapsulation suggests that the permeation-enhancing properties of Soluball^®^ are not limited to a specific molecular class. These findings suggest that Soluball^®^ is a versatile platform capable of delivering diverse hydrophilic cargoes, ranging from low-molecular-weight antioxidants to protein therapeutics, across the skin. Although Strat-M^®^ membranes provide a reproducible and convenient platform for comparative permeation studies, they do not fully reproduce the complex structural organization, lipid composition, appendageal pathways, and biological heterogeneity of native human skin. Therefore, permeation data derived from artificial membranes should be interpreted cautiously and complemented with studies employing biologically relevant skin models. Furthermore, the present study was primarily limited to in vitro and ex vivo experiments, and additional investigations involving in vivo models and mechanistic analyses will be required to further establish the translational applicability of Soluball^®^-based delivery systems. Compared with conventional transdermal carriers, Soluball^®^ provides a highly porous mesoporous architecture suitable for diverse hydrophilic cargos.

### 4.5. Study Limitations

Several limitations of the present study should be acknowledged. Encapsulation efficiency and loading capacity were not quantitatively determined in the present study. Nevertheless, the enhanced transdermal permeation observed for H-S powder indirectly supports successful incorporation of rhHAPLN1 within the mesoporous carrier system. Future investigations should include quantitative evaluation of encapsulation efficiency and loading capacity to further validate the performance of the delivery platform. Controlled release behavior represents an important characteristic of mesoporous delivery systems. However, release kinetics of rhHAPLN1 from Soluball^®^ were not investigated in the present study. Future studies should evaluate sustained release characteristics and release profiles to further elucidate the functionality of the delivery platform. Direct assessment of protein structural integrity following encapsulation was not performed in the present study. Although retained biological activity suggests preservation of rhHAPLN1 functionality, future studies employing circular dichroism spectroscopy, SDS–PAGE analysis, or protein stability assessments would provide additional confirmation of protein integrity after encapsulation. Biological validation in the present study was primarily conducted using HaCaT keratinocytes. Additional biological validation involving oxidative stress markers, apoptosis-related proteins, and inflammatory cytokines was beyond the scope of the present study. Future investigations employing primary human keratinocytes, reconstructed skin models, or in vivo systems, together with evaluation of oxidative stress markers, apoptosis-related proteins, and inflammatory cytokines, may provide a more comprehensive understanding of the biological functions and safety profile of rhHAPLN1, thereby strengthening the translational relevance of the findings. Skin layer distribution, epidermal versus dermal localization, and tissue retention behavior of rhHAPLN1 following transdermal administration were not investigated in the present study. Future studies employing fluorescent imaging, confocal microscopy, or histological analyses may provide further insights into localization patterns and transdermal transport behavior. Therefore, although the present biological findings support the feasibility of the Soluball^®^ platform, these physicochemical limitations should be considered when interpreting the overall performance of the delivery system.

## 5. Conclusions

In this study, rhHAPLN1 effectively preserved both the structural integrity and functional hydrodynamic volume of the hyaluronan-rich pericellular matrix (PCM) in HaCaT keratinocytes under hyaluronidase-induced degradative conditions. In addition, rhHAPLN1 promoted keratinocyte proliferation under serum-free conditions, suggesting a potential role in maintaining epidermal microenvironmental homeostasis. To overcome the limited skin permeability of this hydrophilic biomolecule, we developed Soluball^®^, a dodecylamine-templated mesoporous silica carrier system. Soluball^®^ significantly enhanced the transdermal permeation of rhHAPLN1 across human cadaver skin and also improved the delivery of water-soluble Vitamin C, supporting its broader applicability for hydrophilic cargoes. Collectively, these findings suggest that the combination of rhHAPLN1-mediated PCM stabilization and Soluball^®^-mediated delivery enhancement may represent a promising microenvironment-targeted strategy for regenerative dermatological applications. However, the absence of quantitative encapsulation efficiency, loading capacity, and release kinetics should be considered when interpreting the present findings. Future studies incorporating these physicochemical evaluations together with in vivo validation will further establish the translational potential of the Soluball^®^ delivery platform.

## Data Availability

The data presented in this study are available on request from the corresponding author.

## Abbreviations

The following abbreviations are used in this manuscript

PCM	Pericellular matrix
HA	hyaluronan
rhHAPLN1	Recombinant human hyaluronan and proteoglycan link protein 1
MSNs	Mesoporous silica nanoparticles
SC	Stratum corneum
HAdase	Hyaluronidase
CLSM	Confocal laser scanning microscopy
CCK-8	Cell Counting Kit-8
DLS	Dynamic light scattering
BET	Brunauer–Emmett–Teller
BJH	Barrett–Joyner–Halenda
